# Spectroscopic Studies of Quinobenzothiazine Derivative in Terms of the In Vitro Interaction with Selected Human Plasma Proteins: Part 2

**DOI:** 10.3390/molecules28020698

**Published:** 2023-01-10

**Authors:** Aleksandra Owczarzy, Wojciech Rogóż, Karolina Kulig, Jadwiga Pożycka, Andrzej Zięba, Małgorzata Maciążek-Jurczyk

**Affiliations:** 1 Department of Physical Pharmacy, Faculty of Pharmaceutical Sciences in Sosnowiec, Medical University of Silesia, 40-055 Katowice, Poland; 2 Department of Organic Chemistry, Faculty of Pharmaceutical Sciences in Sosnowiec, Medical University of Silesia, 40-055 Katowice, Poland

**Keywords:** plasma proteins, 5-alkyl-12(H)-quino [3,4-b][1,4]benzothiazinium derivative, fluorescence spectroscopy, UV-vis spectroscopy, circular dichroism

## Abstract

Synthesis of anticancer substances and studying their binding abilities towards human serum proteins as carriers are important parts of pharmaceutical and medical sciences development. The presented work is a continuation of studies of quinobenzothiazine derivatives binding with serum proteins. The main aim of this work was a spectroscopic analysis of second from benzothiazinium derivatives salt, 9-fluoro-5-alkyl-12(H)-quino [3,4-b][1,4]benzothiazinium chloride (Salt2), its interaction with carrier proteins, i.e., human serum albumin (HSA), α_1_-acid glycoprotein (AGP), human gamma globulin (HGG), and the study of protein secondary and tertiary structure changes using spectroscopic techniques (spectrofluorescence, UV-Vis and circular dichroism CD spectroscopy). In order to mimic in vivo conditions, control normal serum (CNS) was used. Using the Klotz method, both binding constants (K_a_ [M^−1^]) and the number of binding classes (n) were calculated. In addition, the percentage of displacement of binding site markers from HSA and AGP molecules has been defined. Based on the obtained data, it can be concluded that the main binding protein for Salt2 is AGP. HSA and HGG are also involved in the distribution of the studied substance in the bloodstream. Moreover, Salt2 very slightly interacts with CNS, which can cause strong therapeutic as well as toxic effects. The analysis of CD spectra confirms that there are no changes in the secondary structure of the main binding proteins in the presence of Salt2.

## 1. Introduction

Cancer diseases are the most frequent cause of death worldwide. Their number is increasing every year and it is so important to develop innovative anticancer drugs that are both highly effective and patient-safe [1]. The synthesis of new drugs, particularly anticancer pharmaceuticals, and the study of their binding abilities towards human serum carrier proteins are important parts of the development of pharmaceutical and medical science [2].

Serum proteins play a fundamental role in the human body. The most abundant protein in the human bloodstream is serum albumin (HSA), which is synthesized by liver cells (hepatocytes). HSA plays numerous functions in the human body, including participation in the transport of exo- and endogenous substances and in the maintenance of proper oncotic pressure or pH. HSA is an important marker of inflammation throughout the body [3,4]. The molar mass of the HSA molecule is approximately 66.5 kDa and consists of a single polypeptide chain of 585 amino acid residues, including only one tryptophanyl residue at position 214 (Trp-214). HSA consists of 35 cysteinyl (Cys) residues, where 34 cysteinyl residues form disulfide bonds that stabilize the tertiary structure. Single cysteinyl residue (Cys-34) is unbound [5]. HSA resembles a heart shape, with approximate dimensions of 80 × 80 × 30 Å [4]. The secondary structure of HSA is dominated by α-helix without β-sheet. Each human serum albumin molecule consists of three homologous domains (I–III), which are similar in secondary and tertiary structure. These domains are divided into two subdomains A and B [6,7]. In molecular structure of HSA, according to Sudlow’s nomenclature, two binding sites I and II can be distinguished, and they correspond to subdomains IIA and IIIA, respectively. The Sudlow site I is characterized by the presence of a single tryptophanyl residue at position 214 (Trp-214) and a positively charged lysine residue. This binding site is mainly nonpolar, but contains two clusters of polar residues: an inner group behind the bottom of the binding pocket (Tyr-150, His-242, Arg-257) and an external region that is located at the entrance to the binding pocket, composed of the following amino acid residues: Lys-195, Lys-199, Arg-218, and Arg-222. Subdomain IIA is limited by residues Trp-214, Leu-219, Phe-223, Leu-238, His-242, Leu-260, Ile-264, Ser-287, Ile-290, and Ala-291. Additionally, in the course of numerous studies, the IIA subdomain has been divided into three regions, Ia, Ib, and Ic, which partially overlap [8,9]. The Sudlow site II corresponds to subdomain IIIA, is smaller and has an elongated form of hydrophobic pocket, with a distinct polar region located near the entrance to the binding pocket. The argininyl residue (Arg-410) and the tyrosyl residue (Tyr-411) are located in subdomain IIIA [9]. Both binding sites I and II play important role in drug distribution in human blood [3,4,7]. 

α_1_-acid glycoprotein (orosomucoid, AGP) is an important protein that is involved in drug distribution in human blood. AGP is a positive acute-phase protein and its concentration level significantly increases during inflammation or therapy [10,11]. AGP is also synthesized in liver and secreted into the bloodstream in small amounts. The concentration of AGP in human serum ranges from 0.6 to 1.2 mg · L^−1^, which corresponds to a range of 1% to 3% of total protein (TP) under physiological conditions [12]. AGP plays an important role in human blood. It takes part in the process of immunomodulation and participates in the transport of basic and neutral endogenous molecules and therapeutic substances [13,14,15]. An α_1_-acid glycoprotein consists of the single polypeptide chain of 183 amino acid residues and five sialylated N-linked glycans. The molar weight of the molecule varies between 41–43 kDa [11]. AGP molecules are built by eight anti-parallel β-strands (βA-βH), connected by four characteristic loops (βA/βB, βC/βD, βE/βF, βG/βH) formed into a β-barrel with three side α-helices (α1, α2, α3). AGP displays up to seven binding sites, but only one plays a significant role in drug binding and its distribution in the human body [16]. This is located inside the β-barrel structure [17]. In the AGP molecules, three tryptophanyl residues appear. The first, Trp-25, is located at the bottom of the β-barrel structure; Trp-122 is located at the entrance to the drug-binding pocket (partially exposed to the aqueous environment); Trp-166 is located on the AGP surface (fully exposed to the environment). In addition, two positively charged arginine residues (Arg-68, Arg-90) are presented at the entrance into the binding pocket and are involved in the binding of negative charged ligands [18]. AGP is encoded by two different genes and is characterized by a high polymorphism, associated additionally with a high heterogeneity of glycans. Due to this fact, different genetic variants may show different affinities for ligands [12]. The major transport protein in human blood serum is HSA, whereas AGP plays a very important role in the binding and distribution of drugs in pathological conditions that significantly involve an increase in AGP levels, for example cancer diseases [2]. 

Gamma globulins (HGGs) are part of immune proteins, and their concentration in the adult serum ranges from 9.5 to 12.5 mg · mL^−1^. HGGs are responsible for regulating immune processes, and are capable of binding various types of antigens, metabolites, organic compounds or drugs. HGGs play an important role in drug-monitored therapy [19]. 

**Figure 1:**
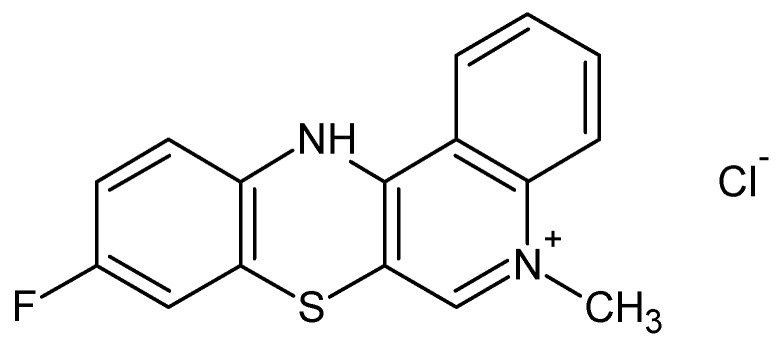
9-Fluoro-5-alkyl-12(H)-quino [3,4-b][1,4]benzothiazinium chloride (Salt2) [20].

The synthesis of anticancer substances, such as quinobenzothiazine derivatives, and the study of their binding abilities towards model carrier plasma proteins represent important parts of the pharmaceutical and medical sciences. Development of 9-fluoro-5-alkyl-12(H)-quino [3,4-b][1,4]benzothiazinium chloride (Salt2) (Figure 1) was conducted by a reaction using thioquine butyrate with the corresponding arylamine groups. The anticancer properties of the newly synthesized compound were tested in vitro on a human colon cancer cell line (HCT 116) and a Lewis Lung carcinoma cell line (LLC). Doxorubicin, the most important drug in chemotherapy, was used as a reference substance. Antiproliferative activity was determined based on drug sensitivity values in terms of inhibitory concentration (IC_50_). Salt2 had activity against both cell lines. In addition, a correlation between the structure of the tested compound and its antiproliferative properties was observed. Quantitative structure activity relationship (QSAR) models were used to determine the correlation between Salt2 activity and its lipophilic parameters. Therefore, 9-fluoro-5-alkyl-12(H)-quino [3,4-b][1,4]benzothiazinium chloride can be considered to be a potential anticancer substance [19,20].

This study is a continuation of quinobenzothiazine derivatives binding with model serum proteins. Analysis of the interaction between ligands and potential carrier proteins using spectrofluorescence and UV–Vis spectroscopy provides a lot of necessary and valuable information. Among other things, it allows us to determine the concentration of the free fraction of the substance (pharmacologically active) in addition to its bound, which is a specific reservoir. The research is of a theoretical and experimental nature, but from a scientific point of view is also novelty due to the promising biological properties of 9-fluoro-5-alkyl-12(H)-quino [3,4-b][1,4]benzothiazinium chloride [21].

## 2. Results and Discussion

To start preclinical and clinical studies of quinobenzothiazine derivatives, it is very important to conduct basic research, involving the analysis of the interaction between quinobenzothiazine salts with model carrier proteins to determine the concentration of compound free and bound fractions. The analysis of the interaction of 9-fluoro-5-alkyl-12(H)-quino [3,4-b][1,4]benzothiazinium chloride (Salt2) with a control normal serum (CNS) is also an important part of the study. This is very significant due to the fact that only free fractions of the drug (those not bound with carrier protein) can cause both therapeutic and toxic effects. In a complementary way, the drug concentration in the target tissue is related to the concentration of unbound drug in human plasma [2].

The analysis of the interaction between the ligand (Salt2) and model serum proteins (HSA, AGP, HGG), the determination of the major transporting protein of the tested compound in bloodstream and the assessment of the characteristic of macromolecules binding sites form the bases for further in vitro and in vivo studies [2,22,23]. Although the conducted research has a basic character, its meaning is innovative and promising from a scientific point of view.

### 2.1. Salt2-HSA, Salt2-AGP, Salt2-HGG, Salt2-CNS Interaction Analysis

The characterization of intermolecular interaction can be possible by analyzing the changes in absorbance of the UV-Vis spectra and in the intensities emission fluorescence spectra of proteins with the increasing ligand concentration. A key role in studies using spectroscopic techniques is played by the aromatic amino acid residues that have the ability to fluoresce. These are mainly tryptophanyl and tyrosyl residues, found in the studied proteins’ macromolecule structure [6,24].

Based on obtained emission fluorescence spectra of human serum albumin (HSA) (Figure S1), α_1_-acid glycoprotein (AGP), human gamma globulin (HGG) and control normal serum (CNS) in the presence of Salt2 with increasing concetration, both at excitation wavelength λ_ex_ 275 nm and λ_ex_ 295 nm (data not shown), the percentage of fluorescence quenching was calculated. Received data have been collected in Table 1.

The data collected in Table 1 show that the strongest quenching of protein fluorescence occurred in the presence of Salt2 with the increasing concentration ranges to 66.07% and 60.88% for AGP, at excitation wavelengths λ_ex_ 275 nm and λ_ex_ 295 nm, respectively. The percentage of human serum albumin (HSA) fluorescence quenching as control serum is similar, reaching about 30% at both excitation wavelengths λ_ex_ 275 nm and λ_ex_ 295 nm. The weakest fluorescence quenching occurs for HGG and equals to 21.25% and 25.07% at excitation wavelengths λ_ex_ 275 nm and λ_ex_ 295 nm, respectively (Table 1). Moreover, the data presented in Table 1 indicate that Salt2 has a higher affinity towards AGP molecules than towards HSA, HGG and control normal serum (CNS) [25].

**Figure 2:**
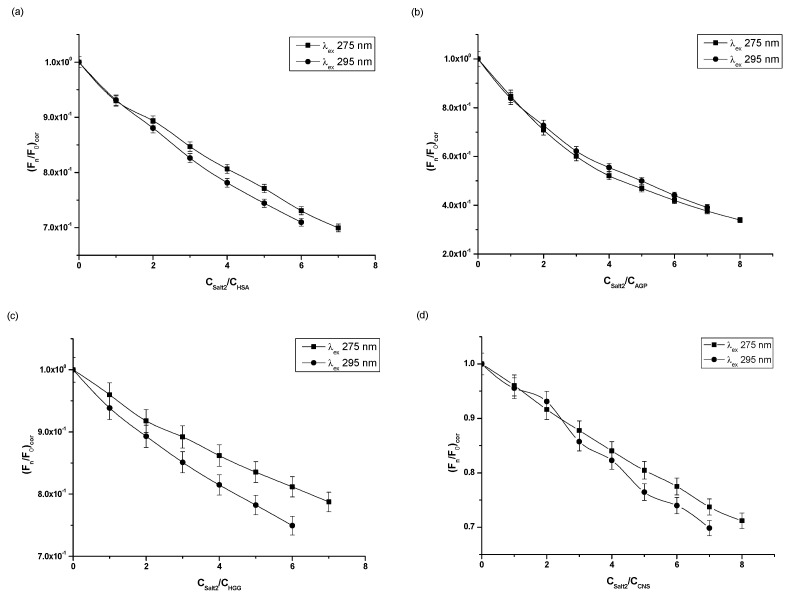
Fluorescence quenching curves of (**a**) HSA, (**b**) AGP, (**c**) HGG, (**d**) CNS at molar concentration 3 × 10^−6^ mol·L^−1^ in the presence of Salt2 at increasing concentration (λ_ex_ 275 nm and λ_ex_ 295 nm).

Based on the fluorescence quenching curves (Figure 2), it was observed that, as the molar ratios Salt2:HSA, Salt2:AGP, Salt2:HGG, Salt2:CNS increased, fluorescence intensity of studied proteins decreased at both excitation wavelengths λ_ex_ 275 nm and λ_ex_ 295 nm. The fluorescence quenching curves of HSA and HGG at a ligand:protein molar ratio from 0:1 to 2:1 overlap (Figure 2a,c). However, the fluorescence quenching curves of control normal serum overlap with molar ratio Salt2:CNS 0:1 to 4:1 (Figure 2d). Above 2:1 (Salt2:HSA, Salt2:HGG) and 4:1 (Salt2:CNS) molar ratios, the course of fluorescence quenching curves is different (Figure 2a,c,d). Meanwhile, fluorescence quenching curves of α_1_-acid glycoprotein both completely overlap at excitation wavelengths λ_ex_ 275 nm and λ_ex_ 295 nm (Figure 2b). The phenomenon of protein fluorescence quenching is associated with the direct energy transfer between Salt2 (acceptor) and the studied protein (donor) if the distance between donor and acceptor is not greater than 10 nm [26]. The same course of fluorescence quenching curves may indicate the participation of only tryptophanyl residues in the interaction between ligand and protein. In contrast, a different trajectory of fluorescence quenching curves indicates the involvement of both tyrosyl and tryptophanyl residues [25]. In addition, the course of fluorescence quenching curves of HSA, AGP, HGG and CNS, in the presence of Salt2 at the excitation wavelength λ_ex_ 275 nm and λ_ex_ 295 nm, allowed us to indicate that the Trp and/or Tyrs fluorophores were involved in the Salt2–protein interaction. The identical course of the fluorescence quenching curves of HSA, HGG and CNS at the molar ratios Salt2:HSA=Salt2:HGG 0:1÷2:1 and Salt2:CNS 0:1÷4:1 (Figure 2a,c,d), at excitation wavelengths λ_ex_ 275 nm and λ_ex_ 295 nm, indicates an initial contribution by only Trps residues in the interaction with Salt2 in the environment of the binding site, with a negligible contribution of Tyrs residues. In contrast, the fluorescence quenching curves of AGP, in the presence of Salt2 with increasing concentrations, overlap. This may indicate that only tryptophanyl residues (Trp-25, Trp-122, Trp-166), present in the AGP molecule, are involved in the interaction between the ligand and protein [18]. In addition, in the case of HSA, the interaction between the HSA molecule and Salt2 takes place mainly in subdomains IIA (Trp-214, Tyr-263) and IIIA (Tyr-401, Tyr-411) [25,26]. Similar fluorescence quenching curves were obtained by Szkudlarek et. al. [27]. They in vitro tested the binding ability of acetohexamide to glycated serum albumin in the presence of fatty acids and confirmed the involvement of individual aromatic amino acid residues in the interaction between the ligand and the proteins.

Based on the recorded emission fluorescence spectra of proteins (data not shown), a decrease in fluorescence intensity in the presence of Salt2 with increasing concentration was observed. This is related to the effect of Salt2 on the tertiary structure of HSA, AGP, HGG and CNS. This effect can also be observed as a result of exposure of tryptophanyl and tyrosyl residues to solvent [28]. In addition, as the concentration of Salt2 increases, a longwave spectrum shift (red shift) of protein was noticed (Table 2). In the presence of Salt2, slight long-term spectrum shifts (red shift) of AGP at excitation wavelength λ_ex_ 275 nm (Δλ_max_ 2 nm) and of CNS at excitation wavelength λ_ex_ 295 nm (Δλ_max_ 2 nm) were observed and remain within the limits of measurement uncertainty. Due to the sensitivity to small changes in the position of protein maximum fluorescence wavelength (λ_max_) [29], spectral parameters A ($A=\frac{F_{365\;\mathrm{nm}}}{F_{320\;\mathrm{nm}}}$) have been calculated and data were collected in Table 2. 

Studies conducted by Maciążek-Jurczyk et al. [29], involving the evaluation of the effect of oxidative stress on the structure of HSA as a carrier protein of the diazaphenothiazine with potential anticancer activity, also proved that spectral parameter A allows us to determine the changes in protein structure. Due to the high absorbance of Salt2 at wavelength λ_max_ 303.6 nm (data not shown), it was not possible to calculate full width at half maximum (FWHM). Spectral parameter A has also been used by Parkhomenko et al. in spectrofluorimetric analysis of albumin preparations from healthy donors and patients with kidney disease. This parameter A was used to evaluate the maximum fluorescence intensity shifts and thus to assess changes in the tertiary structure of HSA [30]. Similar to the previous study [2], an increase in the values of calculated spectral parameters A was observed for Salt2-HSA, Salt2-AGP, Salt2-HGG and Salt2-CNS. An increase in the values of the parameter A indicates an increase the hydrophilicity of the environment around tyrosyl and tryptophanyl residues and their exposure to the external environment. Based on the data collected in Table 2 and presented in our previous work [2], it can be assumed that Salt2 changes the environment around tyrosyl and tryptophanyl residues to be more hydrophilic and therefore is a factor that can modify the tertiary structure of HSA, AGP, HGG and also proteins contained in the control normal serum (CNS). This phenomenon also approves the existence of interaction between the protein molecules and the Salt2, as well as the interaction of the ligand with control normal serum.

**Figure 3:**
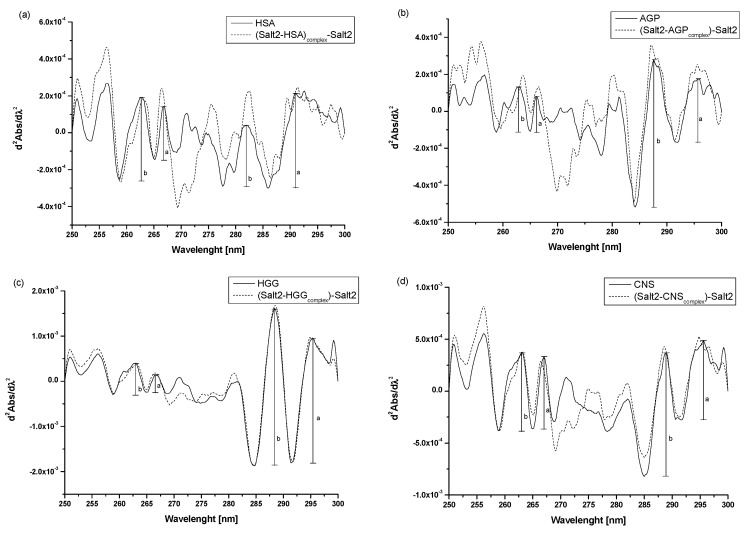
Second derivative absorption spectra of (**a**) HSA vs. (Salt2-HSA_complex_)-Salt2, (**b**) AGP vs. (Salt2-AGP_complex_)-Salt2, (**c**) HGG vs. (Salt2-HGG_complex_)-Salt2, (**d**) CNS vs. (Salt2-CNS_complex_)-Salt2 after subtraction of Salt2 absorption, respectively. [Proteins concentration] 3 × 10^−6^ mol·L^−1^; [Salt2]:[HSA] 7:1 molar ratio, [Salt2]:[AGP] 8:1 molar ratio, [Salt2]:[HGG] 7:1 molar ratio, [Salt2]:[CNS] 7:1 molar ratio; a, b—peaks height.

Second derivative spectroscopy of differential spectra allows us to observe the detection of small changes in spectral features, such as arms and ripples, and to classify them with very small or negligible errors [31]. Thus, it is a useful additional method for assessing environmental changes around aromatic amino acid residues. The spectra of the second derivative of the differential absorption spectra at the wavelength range from 250 nm to 270 nm reveal environmental changes around phenylalanyl residues, while those above 270 show changes within tyrosyl and tryptophanyl residues [31,32,33]. To confirm the effect of Salt2 on the tertiary structure of the main carrier proteins, second derivatives of the differential absorption spectra were recorded (Figure 3a–d).

Analyzing the obtained spectra, it is possible to observe the changes in their course due to the presence of Salt2. These include increases or decreases in the absorbance of the studied proteins and the transformation of arms into distinct peaks. According to the previous studies [2], r parameters ($r=\frac{a}{b}$, see Figure 3) were calculated to confirm the changes in the surroundings of phenylalanyl, in tyrosyl/tryptophanyl residues of HSA, AGP, HGG, as well as in CNS (Table 3).

Ichikawa et al. [31] used the method of differential absorption spectra second derivative to evaluate the effect of denaturing agents on protein environment and the content of phenylalanyl residues in macromolecules. Moreover, Tereda et al. [34] evaluated the impact of the system’s external factors on tyrosyl and tryptophanyl residues in the studied proteins. Thus, it can be concluded that second derivative spectroscopy of differential absorption spectra is a useful method to detect and confirm microenvironment changes around aromatic amino residues. Similar to previous study conducted by Owczarzy et al. [2], an analysis of the changes in the value of r parameter confirms that the effect of Salt2 on the tertiary structure of HSA, AGP, HGG and CNS was the same as for Salt1 and the change in the microenvironment around the aromatic amino acid residues. Namely, it became more hydrophilic. Based on this, it can be concluded that the presence of a fluorine substituent at position 9 of Salt2 does not significantly affect the environmental changes around the aromatic amino acid residues of the tested proteins.

**Figure 4:**
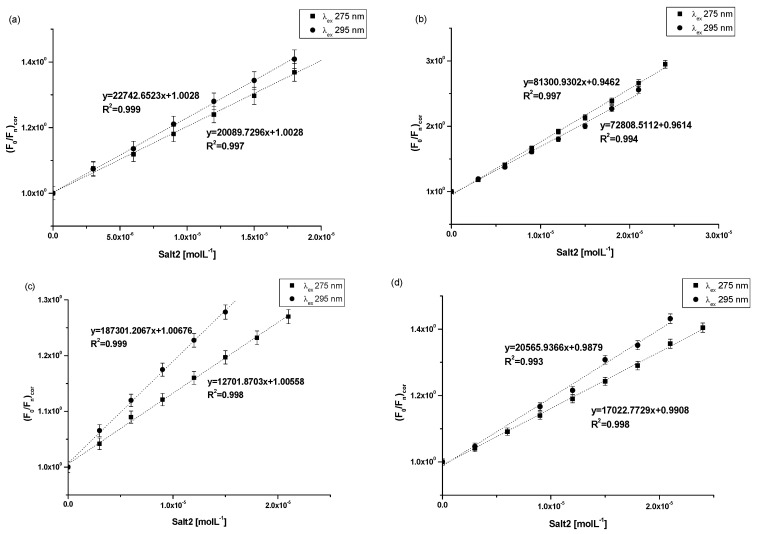
Stern–Volmer plots for (**a**) 3 × 10^−6^ mol·L^−1^ HAS, (**b**) 3 × 10^−6^ mol·L^−1^ AGP, (**c**) 3 × 10^−6^ mol·L^−1^ HGG and (**d**) 3 × 10^−6^ mol·L^−1^ CNS in the presence of Salt2 at increasing concentration (λ_ex_ 275 nm and λ_ex_ 295 nm).

An additional, useful method for characterizing ligand–protein interaction is fluorescence quenching. Fluorescence quenching is an important method for measuring binding affinity between ligands and proteins and this phenomenon is observed in donor–acceptor systems when the excited fluorophores present in the protein molecule are deactivated. The quenching process, which results in a decrease in fluorescence intensity, could have a different nature: static, dynamic or mixed (static–dynamic). Static quenching is caused by the formation of a donor–acceptor complex in the ground state, which does not exhibit the ability to fluoresce. The second type of quenching is associated with energy transfer as a result of collisions of molecules present in the system. During the contact of the chromophore with the fluorophore which is in the excited state, a transfer of energy takes place, as a result of which the donor returns to the ground state [35,36,37]. To determine the type of fluorescence quenching of the studied proteins in the presence of increasing ligand concentration, the Stern–Volmer equation (Equation (3)) was used and the Stern–Volmer curves were presented on Figure 4.

At both excitation wavelengths (λ_ex_ 275 nm and λ_ex_ 295 nm), the Stern–Volmer curves for Salt2-HSA, Salt2-AGP, Salt2-HGG and Salt2-CNS systems in the presence of Salt2 with increasing concentration are linear (Figure 4a–d). Therefore, the Stern–Volmer constants (K_S-V_) and the bimolecular fluorescence quenching constants rate (k_q_) were determined and collected in Table 4.

The Stern–Volmer constant (K_S-V_) allows us to determine the distance between the excited fluorophore and the ligand. With the increase in K_S-V_ value, a smaller distance is obtained and the complex becomes stronger. A stronger complex means that the therapeutic effect is weaker [28,38]. Based on the data collected in the Table 4, the highest values of Stern–Volmer constants (K_S-V_) were observed for the Salt2-AGP system (K_S-V_ equals to 8.13 ± 0.15 × 10^4^ mol·L^−1^ and 7.28 ± 2.13 × 10^4^ mol·L^−1^ at λ_ex_ 275 and 295 nm, respectively) compared to the other studied systems at both excitation wavelengths. Similarly, as in the previous work [2], higher values of Stern–Volmer constants (K_S-V_) were also obtained for the Salt1-AGP complex than for other proteins (K_S-V_ equals to 5.40 ± 0.10 × 10^4^ mol·L^−1^ and 6.72 ± 0.10 × 10^4^ mol·L^−1^ at λ_ex_ 275 and 295 nm, respectively). This suggests that Salt1 and Salt2 have the strongest affinity towards excited fluorophores of α_1_-acid glycoprotein, and registers the shortest distance between the ligand and the excited fluorophores registered, whereas Salt2 has a higher affinity than Salt1. Based on the K_S-V_ constants values it can be concluded that Salt2 forms a stronger system with AGP than Salt1, or that Salt2 interacts with this protein in several low- and high-affinity binding sites. This phenomenon may be related to the presence of the fluorine substituent at position 9 of the Salt2 structural formula [2,20]. Due to the linear course of Stern–Volmer curves for Salt2-HSA, Salt2-AGP, Salt2-HGG and Salt2-CNS systems at both λ_ex_ 275 nm and λ_ex_ 295 nm (R^2^ correlation coefficient equals to 0.99), it was not possible to clearly determine the character of the interaction occurring between the studied proteins and Salt2 (Figure 4a–d). Therefore, based on the Stern–Volmer equation (Equation (3)), the bimolecular quenching constant rate (k_q_) was calculated and a unambiguous assessment of the character of fluorescence quenching of the studied protein was performed. For dynamic (collisional) fluorescence quenching, according to Lakowicz, the maximum value of k_q_ in aqueous solution is equal to 1 × 10^10^ mol^−1^·L·s^−1^ [35]. Based on the data collected in Table 4, it can be concluded that the quenching of HSA, AGP, HGG, CNS fluorescence is static, since the obtained k_q_ values are of the order of 10^12^. According to the work of Van de Weert and Stella [37], this may indicate that Salt2 inhibits the formation of the excited state of the fluorophores present in the carrier proteins. The intensity of the emitted fluorescence of the studied proteins, in the presence of Salt2 with increasing concentration, works to decrease simultaneously the population of available and excited fluorophores.

**Figure 5:**
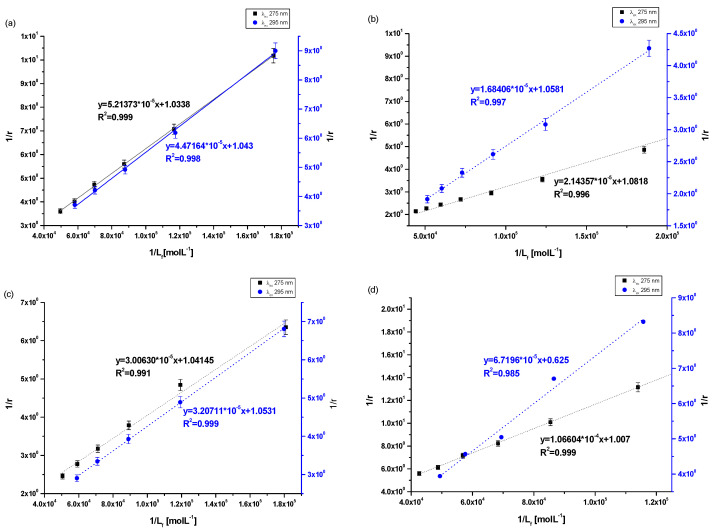
The Klotz plots for (**a**) Salt2-HSA; λ_ex_ 275 nm, λ_ex_ 295, (**b**) Salt2-AGP; λ_ex_ 275 nm, λ_ex_ 295, (**c**) Salt2-HGG; λ_ex_ 275 nm, λ_ex_ 295, (**d**) Salt2-CNS; λ_ex_ 275 nm, λ_ex_ 295 nm.

The association constant (K_a_) characterizes the stability of the formed complex. In order to determine the association constants (K_a_) for Salt2 systems with HSA, AGP, HGG and CNS, and to identify the number site of binding classes (n) based on the Klotz equation (Equation (4)), Klotz curves were plotted (Figure 5a–d) and K_a_ values were calculated and collected in Table 5.

The ability to bind therapeutic substances by carrier serum proteins is the basis for modulation at the target site in the effectiveness of the therapeutic substance concentration according to the free drug theory, which assumes that only the free fraction of the drug has a therapeutic effect. Once the equilibrium is established, the concentration of the free drug in serum or whole blood is equal to the concentration of the drug at the target site [39].

Analyzing the value of the association constant (K_a_) obtained by the Klotz method (Figure 5a–d, Table 5), it was found that the highest value of K_a_ was calculated for (Salt2-AGP)_complex_ and equals to 5.04 ± 0.25 × 10^4^ mol·L^−1^ and 6.28 ± 0.11 × 10^4^ mol·L^−1^ at excitation wavelength λ_ex_ 275 nm and 295 nm, respectively. Salt2 forms with α_1_-acid glycoprotein the strongest complex. Due to the basic character of AGP, it is the main binding protein of Salt2 in the human bloodstream [40]. Salt2 forms complexes with HSA and HGG but with lower values of association constant than with AGP, implying weaker (Salt2-HSA)_complex_ and (Salt2-HGG)_complex._ Nevertheless, HSA and HGG are participating in the distribution of Salt2 in the bloodstream. Based on this, it can be assumed that Salt2 can be relatively easier released from the complex with HSA, then with HGG. In contrast, AGP may be a specific reservoir of 5-alkyl-12(H)-quino [3,4-b][1,4]benzothiazinium salts (Salt1, Salt2) in human blood [40]. Due to the variable concentration of α_1_-acid, glycoprotein plays an important role in the binding and transporting of basic drugs, depending on the condition of the body (AGP concentration under physiological conditions is in the range between 0.6 and 1.2 mg · L^−1^, and increases two or three times in pathological states especially in cancer diseases) [12]. Thus, it can be expected that when the serum AGP concentration in human serum is low, Salt2 distribution can occur via HSA and HGG molecules. In addition, high concentrations of α_1_-acid glycoprotein, as a result of cancer-related inflammation, may elongate the effect of Salt2. This is a very positive phenomenon, because it can allow the personalization of the patient’s dose and thus minimize the side effects of therapy [23,40,41].

According to free drug theory, it is extremely important to determine the transport capacity of blood serum, which is a mixture of all transport proteins and other non-morphotic blood elements. For this purpose, control normal serum (CNS) was used and complexation parameters were determined [39]. In control normal serum, the percentage of HSA about 80% while HGG ranged from 8.1 to 19.9%. The values of the association constants (K_a_) for the (Salt2-CNS)_complex_ are identical for both excitation wavelengths λ_ex_ 275 nm and λ_ex_ 295 nm and equal to 0.94 ± 0.04 × 10^4^ mol·L^−1^ and 0.91 ± 0.43 × 10^4^ mol·L^−1^, respectively. The low values of obtained association constants confirm the interaction of CNS with Salt2 and/or the formation of the Salt2-CNS complex. This may affect both strong therapeutic and toxic effects, further confirming the need to select the dose of the test substance according to individual patient needs, based on the parameters of the protein profile. It is also necessary to monitor the concentration of the drug in order to avoid exceeding the therapeutic dose and side effects of therapy. Until now, 9-fluoro-5-alkyl-12(H)-quino [3,4-b][1,4]benzothiazinium chloride (Salt2) has not been tested as a substance transported by HSA, AGP, HGG and control normal serum, and from the scientific point of view these studies are novelty. Studies which were conducted previously [2] using 5-methyl-12(H)-quino [3,4-b]-1,4-benzothiazinium chloride (Salt1), a derivative of 9-fluoro-5-alkyl-12(H)-quino [3,4-b][1,4]benzothiazinium chloride (Salt2), allowed us to determine the character of interaction and to calculate association constants for HSA, AGP, HGG and CNS of the same order (10^4^ mol·L^−1^). Thus, it was confirmed that the main carrier protein of the studied substances with potential anticancer activity is α_1_-acid glycoprotein. The similar affinity of both Salt1 and Salt2 for human serum albumin and control normal serum was also confirmed. In contrast, Salt2 forms a slightly stronger complex with HGG (K_a_ equals to 3.45 ± 0.29 × 10^4^ mol·L^−1^ and 3.28 ± 0.10 × 10^4^ mol·L^−1^ at excitation wavelength λ_ex_ 275 nm and 295 nm, respectively) than Salt1 (K_a_ equals to 1.60 ± 0.03 × 10^4^ mol·L^−1^ and 1.14 ± 0.04 × 10^4^ mol·L^−1^ at excitation wavelength λ_ex_ 275 nm and 295 nm, respectively). On this basis, it can be speculated that the presence of a fluorine substituent at position 9 in the structural formula (Salt2) does not affect the ability of Salt2 to bind to major plasma carrier proteins (HSA, AGP), while it has an insignificant impact on the strength of binding to HGG.

**Figure 6:**
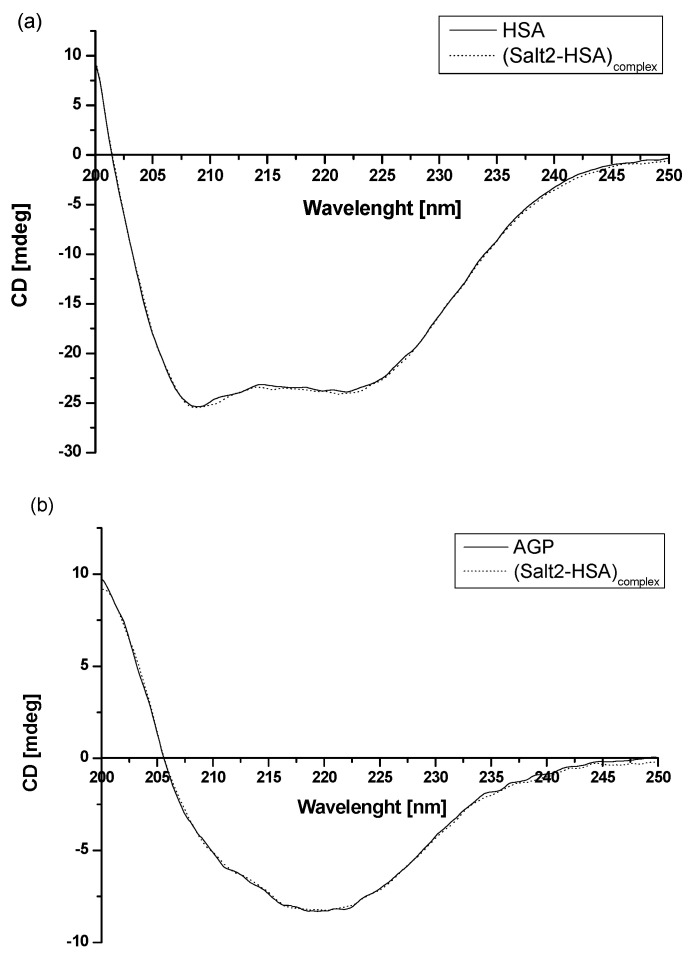
Far-UV circular dichroism (CD) spectra of (**a**) 2 × 10^−6^ mol·L^−1^ HSA and (**b**) 2 × 10^−6^ mol·L^−1^ AGP in the absence and presence of Salt2. [Salt2]:[HSA]=[Salt2]:[AGP] 4:1 molar ratio.

Based on the data obtained from Figure 2 and presented in Table 1, it can be concluded that human serum albumin and α_1_-acid glycoprotein can be treated as the main blood carrier proteins for Salt2. To evaluate how the presence of Salt2 has affected the secondary structure of HSA and AGP, circular dichroism (CD) spectroscopy was used (Figure 6). CD spectroscopy is one of the most useful techniques for assessing the secondary structure of proteins. In addition, it also allows us to determine the protein folding and binding properties and can be used to study ligand–protein interactions [42].

The observed HSA and AGP ellipticity ([mdeg]) illustrate that HSA is an α-helical protein with two characterized bands at λ_min_ 210 nm and λ_min_ 220 nm [43]. In contrast, AGP is an example of a protein with a dominant β-sheet structure. It is characterized by one negative band at λ_min_ 222 nm [37]. Based on recorded spectra (Figure 4), it can be concluded that the intensity band of HSA and AGP in presence of Salt2 ([Salt2]:[HSA]=[Salt2]:[AGP] 4:1 molar ratio) does not change. 

The percentage (%) content of HSA and AGP secondary structure elements was obtained using a secondary structure estimation program with Yang’s and Reed’s reference models, respectively, and data were presented in Table 4. Different reference models were used due to the better fit of the curves: unknown and calculated. In addition, based on equation (Equation (5)), the mean residue ellipticity [Θ_MRW_], for both HSA and AGP, was calculated and presented in Table 6.

Using CD spectroscopy, Munro et al. [44] conducted studies involving structural analysis of the interaction of cytochrome P-450 and BM3 domains. The far-UV spectra overlapped for both purified P-450 reductions of domains and mixtures of domains. Based on this, it was deduced that the connection of two main domains is not accompanied by a change in secondary structure, and that circular dichroism spectroscopy is a useful technique for this type of study. In our previous study, in order to analyze the binding capacity of 5-methyl-12(H)-quino [3,4-b][1,4]benzothiazinium chloride (Salt1) and the interaction with potential carrier proteins, far UV-Vis spectra were also registered [2]. Similarly, as in the previous work, Salt2 does not affect the secondary structure of HSA and does not influence HSA α-helix and β-sheet contents or AGP secondary structure. Moreover, it can be speculated that, although the fluorine substituent is located at position 9, it causes the Salt2 to interact with HSA and AGP and does not destabilize their secondary structure. This is clinically relevant because disruption of the secondary structure of proteins can lead to the modification of their biological functions [45,46]. 

### 2.2. Salt2–Protein Binding Sites Assessment

In the structure of the human serum albumin molecule, two binding sites with known structure and high affinity for binding the drug, called Sudlow sites, were identified. Sudlow site I corresponds to subdomain IIA, while Sudlow site II corresponds to subdomain IIIA. Both are located in the hydrophobic cavities of the HSA molecule. Sudlow sites I and II have completely different shape, size and drug-binding capacity, depending on their polarity [47]. In order to determine the binding sites of Salt2 in HSA molecule, dansylated amino acids were used. Dansylated amino acids are located in the specific albumin molecule binding sites and are characterized by fluorescent activity. The binding ability of dansylated amino acids results from its structure. Amino acids, carrying an electric charge or having a polar side chain, are characteristic of the IIA subdomain, while those having a hydrophobic side chain in their structure are characteristic of the IIIA subdomain. Dansyl-L-glycine (dGly) and dansyl-L-phenylalanine (dPhe) were used to determine binding sites in the HSA molecule. dGly binds to Sudlow site I, while dPhe binds to the Sudlow site II [48,49,50].

An α_1_-acid glycoprotein (AGP) is an acidic protein with a positive electrical charge. AGP has the shape like a β-barrel, surrounded by an α-helix which forms a pocket for ligands. The geometry of the ligand-binding pocket is very complex. The ligand-binding gap is formed by three lobes: (main lobe I)-large and non-polar. On the lobe I sites, there are two smaller and negatively charged lobes II and III. The third lobe also has a small additional entrance [51]. Literature data show that the AGP molecule contains up to seven binding sites with different properties, but that only one of them have clinically relevant properties [52]. To confirm the connection of the binding site of Salt2 to the AGP molecule, quinaldine red (2-[4-(dimethylaminostyryl]-1-ethylquinolones), QR) was used. QR is a specific fluorescent marker for the orosomucoid (AGP) [52]. Based on the obtained fluorescence emission spectra, the percentage of displacement of dansylated amino acids from the HSA molecule and of QR from the AGP molecule were calculated in relation to the increasing concentration of Salt2 (Equation (1)) and collected in Table 7 and Table 8:

(1) $$\mathrm{percentage}\;\mathrm{of}\;\mathrm{displacement}=\frac{F_{0}-F}{F_{0}}\times 100\;\%$$

where: F_0_, F—fluorescence of the marker in the system with protein and both with protein and ligand, respectively

Based on the data collected in Table 7 and Figure 2, it can be concluded that Salt2 displaces both dGly and dPhe from their binding sites in the HSA molecule. At the same time, a higher percentage of displacement was obtained for dGly than for dPhe. Therefore, based on Table 7 as well as Figure 2, it can be speculated that Salt2 first binds to subdomain IIA, which is the main binding site with high affinity to the ligand, and then to subdomain IIIA. Previous studies have proved [2] that Salt1 displaces fluorescent markers from both binding sites in the HSA molecule with the same potency, further confirming that Salt1 has two binding sites with a high affinity for HSA in contrast to Salt2 [2]. Due to this fact, Salt2 may be a more beneficial choice for multidrug therapy.

Based on the obtained data collected in Table 8, it can be concluded that Salt2 displaces quinaldine red with the same intensity at both molar ratios [AGP]:[QR] 1:1 and 1:0.5, meaning that the Salt2 binding site overlaps with the QR binding site in the AGP molecule [51]. Comparing the values of percentages displacement obtained for Salt1 in the previous studies with those obtained for Salt2, it can be concluded that Salt2 displaces QR more strongly from the AGP molecule [2].

The differences in percentage values of fluorescent marker displacement, in the presence of Salt1 and Salt2 with increasing concentrations for both HSA and AGP molecules, may be due to the presence of a fluorine substituent at position 9 in the Salt2 structure [2,20,21].

## 3. Materials and Methods

Human serum albumin, fraction V, Lot No 2,742,726 (HSA), dansyl-L-phenylalanine, Lot No 8776KA (dPhe) were purchased from MP Biomedicals, Inc. (Illkirch, France). Human gamma globulin, Lot No 268129/1 32,705,352 (HGG), was obtained from Fluka Chemie AG (Buchs, Switzerland). α_1_-acid glycoprotein, Lot No 049K7565V (AGP), dansyl-glycine, Lot No 9,143,321 (dGly), quinaldine red, Lot No MKBD6820 (QR) and methanol, Lot No SHBG8324V were gained from SIGMA-ALDRICH Chemie GmbH (St. Louis, MO, USA) while control normal serum (CNS), Lot 200054/724 has been obtained from Alpha Diagnostic (Warszawa, Poland). 9-fluoro-5-alkyl-12(H)-quino [3,4-b][1,4]benzothiazinium chloride (Salt2) has been synthesized in the Department of Organic Chemistry, Faculty of Pharmaceutical Sciences in Sosnowiec, Medical University of Silesia in Katowice, Poland, according to described procedure [21,22].

### 3.1. Methods

#### 3.1.1. Sample Preparation

Based on the previous studies [2], human serum albumin (HSA) solutions at 2 × 10^−6^ mol·L^−1^, 3 × 10^−6^ mol·L^−1^, 5 × 10^−6^ mol·L^−1^ concentrations and α_1_-acid glycoprotein (AGP), human gamma globulin (HGG), control normal serum (CNS) at 2 × 10^−6^ mol·L^−1^ and 3 × 10^−6^ mol·L^−1^, concentrations, respectively, were incubated at 298 K in 0.05 mol·L^−1^ phosphate buffer at pH 7.4. A stock solution of 9-fluoro-5-alkyl-12(H)-quino [3,4-b][1,4]benzothiazinium chloride (Salt2) at 3 × 10^−3^ mol·L^−1^ concentration and dansyl-glycine (dGly), dansyl-l-phenylalanine (dPhe) at 2.5 × 10^−3^ mol·L^−1^ and quinaldine red (QR) at 3 × 10^−3^ mol·L^−1^ concentrations were prepared in methanol. Ligand–protein binding measurements have been conducted at Salt2:HSA 0:1-7:1, Salt2:AGP 0:1-8:1, Salt2:HGG 0:1-7:1 and Salt2:CNS 0:1-8:1 molar ratios at excitation wavelength λ_ex_ 275 nm and Salt2:HSA 0:1-6:1, Salt2:AGP 0:1-7:1, Salt2:HGG 0:1-6:1 and Salt2:CNS 0:1-7:1 molar ratios at excitation wavelength λ_ex_ 295 nm. For the binding sites assessments, HSA and AGP solutions, both in the absence and presence of fluorescent probes at HSA:dPhe 1:1 and HSA:dGly 1:1 molar ratios were titrated by Salt2 at 3 × 10^−6^ mol·L^−1^–3.3 × 10^−5^ mol·L^−1^ concentrations while at AGP:QR 1:0.5 and 1:1 molar ratios were titrated by Salt2 at 3 × 10^−6^ mol·L^−1^–4.8 × 10^−5^ mol·L^−1^ concentrations. To determine changes in proteins secondary structure in complexes Salt2:HSA and Salt2:AGP 4:1 molar ratio has been used.

#### 3.1.2. Emission and Absorption Spectra Measurements

The fluorescence measurements were recorded at 298 K using fluorescence spectrophotometer JASCO FP-6500 with quartz cells at 10 mm path length. Accuracy of wavelength was ±1.5 nm. Emission fluorescence spectra of proteins in the presence of Salt2 (Salt2-HSA, Salt2-AGP, Salt2-HGG, Salt2-CNS) were recorded using λ_ex_ 275 nm and λ_ex_ 295 nm excitation while emission spectra of fluorescent probes both in the absence (HSA-dPhe, HSA-dGly) and titrated by Salt2 were obtained using λ_ex_ 350 nm excitation. For AGP-QR complexes, in the absence and presence of Salt2, λ_ex_ 500 nm excitation has been used. The scattering spectrum of solvent (phosphate buffer) has been subtracted from all the spectra. 

Due to the absorption of light at both excitation and emission wavelengths (inner filter effect, IFE), a correction of Salt2–proteins systems fluorescence intensity is required. Using a JASCO V-530 spectrophotometer, the absorbance measurements at the wavelength used to excite fluorophores fluorescence and at emission wavelength as well as the absorbance measurements for second derivative of differential spectra in the range between 250 nm and 300 nm were made [31]. For the inner filter correction equation (Equation (2)) has been used [31,47]. This equation can be used as long as the absorbance increase in the system is not greater than ≈0.3:

(2) $$F_{\mathrm{cor}}=F_{\mathrm{obs}}\times {\;e}^{\frac{\mathrm{Aex}+\mathrm{Aem}}{2}}$$

where:

F_cor_ and F_obs_—corrected and observed fluorescence (after subtraction the solvent scattering spectrum), respectively,
A_ex_ and A_em_—the absorbance at the excitation and emission wavelength, respectively.

The fluorescence quenching effect (static and/or dynamic) of HSA, AGP, HGG, CNS, both in absence and presence of Salt2, has been analyzed based on the Stern–Volmer equation (Equation (3)) [47]:

(3) $$\frac{F_{0}}{F}=1+k_{q}\tau_{0}\times \left[L\right]=1+K_{\mathrm{SV}}\times \left[L\right]$$

where:

F, F_0_—the fluorescence intensities at the maximum wavelength of albumin in the presence and absence of a quencher, respectively,
$k_{q}=\frac{K_{\mathrm{SV}}}{\tau_{0}}$—bimolecular quenching rate constant in mol^−1^·L·s^−1^,
τ_0_—the average fluorescence lifetime of protein without quencher (τ_0 HSA_ = 6.000 × 10^−9^ s [36], τ_0 AGP_ = 2.285 × 10^−9^ s [18], τ_0 HGG_ = τ_0 CNS_ = 1.000 × 10^−8^ s),
[L]—ligand concentration in mol·L^−1^ ([L] = [L_b_] + [L_f_], where [L_b_] and [L_f_] are the bound and unbound (free) drug concentrations, respectively),
K_S-V_—Stern–Volmer constant in mol^−1^·L.

The association constant (K_a_) in ligand–protein systems has been determined by the Klotz equation (Equation (4)) [53]:

(4) $$\frac{1}{r}=\frac{1}{n}+\frac{1}{n\cdot K_{a}\cdot \left[L_{f}\right]}$$

where:

r—number of ligand moles bound to 1 mole of protein; $r=\frac{L_{b}}{\left[P\right]}$, $L_{b}=\frac{\Delta F}{\Delta F_{\max}}\times P_{t}$,
n—number of binding sites classes,
K_a_—association constant in mol^−1^·L,
[L_f_]—free ligand concentration in mol·L^−1^.

#### 3.1.3. Circular Dichroism (CD) Measurements

Far UV-CD spectra of HSA and AGP were recorded using a JASCO J-1500 CD spectropolarimeter, equipped with a thermostatic Peltier cell holder with an accuracy of ±0.05 °C. Circular dichroism measurements were made in a nitrogen atmosphere at 298K in 1 mm path length quartz cuvette. Samples were scanned from 200 nm to 250 nm at wavelength intervals of 0.2 nm. Prior to the calculation of the final ellipticity, CD protein spectra were corrected by subtraction of spectra obtained for the phosphate buffer, pH 7.4 ± 0.1, measured under identical conditions. Then, using the Savitzky and Golay filters method and 13 convolution width, the obtained spectra were smoothed. CD intensity is expressed as mean residue ellipticity at wavelength λ ([θ]_mre_) according to the equation (Equation (5)) [54,55]:

(5) $${\left[\theta \right]}_{\mathrm{mre}}=\frac{\mathrm{MRW}\;\times {\;\theta }_{\lambda }}{10\times l\;\times \;c}\;\left[\deg\cdot {\mathrm{cm}}^{2}\cdot {\mathrm{dmol}}^{-1}\right]$$

where:

MRW—mean residue weight (MRW_HSA_ = 113.7 Da; MRW_AGP_ = 236.3 Da),
θ_λ_—observed ellipticity at wavelength λ in deg,
l—optical path length in cm,
c—protein concentration in g·cm^−3^.

### 3.2. Statistics

The results of the study were expressed as a mean ± relative standard deviation (SD) from three independent experiments. Linear regression (R^2^) was analyzed using OriginPro version 8.5 SR1 software (Northampton, MA, USA) by fitting experimental data to the corresponding equation.

## 4. Conclusions

The main aim of this project was to analyze a quinobenzothiazine derivative (9-fluoro-5-alkyl-12(H)-quino [3,4-b][1,4]benzothiazinium chloride, Salt2) with anticancer potential in terms of the interaction with main carrier proteins in human blood (HSA, AGP, HGG) and control normal serum (CNS), which mimics human serum, using spectroscopic techniques (spectrofluorescence, UV-Vis and circular dichroism (CD) spectroscopy). In addition, the effect of the fluorine substituent present at position 9 in the Salt2 molecule was compared with the 5-methyl-12(H)-quino [3,4-b]-1,4-benzothiazinium chloride (Salt1) developed in previous studies. During Salt1–proteins and Salt2–proteins complexes formation, the environment of amino acids residues taking part in the interaction becomes more hydrophilic and more polar. The qualitative analysis provided the information that the main binding protein for Salt1 and Salt2 is α_1_-acid glycoprotein (AGP), whereas Salt2 binds slightly weaker with AGP than Salt1. This may be related to the presence of a fluorine substituent at position 9 in Salt2 structure that has a quantifiable effect on the Salt2 distribution in the bloodstream. It is noteworthy that both human serum albumin (HSA) and also human gamma globulin (HGG) take part in Salt2 distribution in human body, but HGG shows a higher proportion of interaction than with Salt1. Using control natural serum (CNS), which is a mixture of all transport proteins found in the human bloodstream, the ability to distribute Salt2 in the bloodstream was confirmed. It is worth noting that changes/modifications in the structure due to the presence of a fluorine substituent at position 9 of Salt2 may contribute to increased/decreased binding capacity to carrier proteins. It may affect the distribution of the compound in the human body. Significantly noticeable differences, compared to Salt1, were obtained for the Salt2-CNS complex. This can strongly affect both the therapeutic and toxic properties of Salt2. The research is of theoretical and experimental nature, but is novelty from a scientific point of view due to the promising biological properties of 9-fluoro-5-alkyl-12(H)-quino [3,4-b][1,4]benzothiazinium chloride. They can also form the basis for further preclinical and clinical studies.

## Figures and Tables

**Table 1:** Percentage [%] of fluorescence quenching of HSA, AGP, HGG and CNS in the presence of Salt2 with increasing concentration at excitation wavelenght λ_ex_ 275 nm and λ_ex_ 295 nm.

Salt2:protein Molar Ratio	λ_ex_ 275 nm		λ_ex_ 295 nm	
	Molar Ratio	Percent of Fluorescence Quenching [%]	Molar Ratio	Percent of Fluorescence Quenching [%]
Salt2:HSA	0:1 ÷ 7:1	30.03	0:1 ÷ 6:1	29.02
Salt2:AGP	0:1 ÷ 8:1	66.07	0:1 ÷ 7:1	60.88
Salt2:HGG	0:1 ÷ 7:1	21.25	0:1 ÷ 6:1	25.07
Salt2:CNS	0:1 ÷ 7:1	28.79	0:1 ÷ 6:1	30.14

**Table 2:** Fluorescence parameters of HSA, AGP, HGG and CNS at 3 × 10^−6^ mol·L^−1^ concentrations (λ_ex_ 275 nm and λ_ex_ 295 nm).

Protein	275 nm			295 nm		
	Salt2 [mol·L^−1^]	λ_max_ [nm]	Parameter A	Salt2 [mol·L^−1^]	λ_max_ [nm]	Parameter A
HSA	0	334	0.80	0	339	1.15
	2.1 × 10^−5^	337	0.90	1.8 × 10^−5^	343	1.32
AGP	0	331	0.61	0	333	0.64
	2.4 × 10^−5^	333	0.67	2.1 × 10^−5^	337	0.77
HGG	0	331	0.61	0	332	0.67
	2.1 × 10^−5^	334	0.72	1.8 × 10^−5^	335	0.79
CNS	0	331	0.60	0	335	0.83
	2.4 × 10^−5^	334	0.76	2.1 × 10^−5^	337	0.93

**Table 3:** r values, calculated from the second derivatives differential absorption spectra of phenylalanyl, tyrosyl/tryptophanyl residues of HSA, AGP, HGG and CNS in the presence of Salt2; [Salt2]:[HSA] 7:1 molar ratio, [Salt2]:[AGP] 8:1 molar ratio, [Salt2]:[HGG] 7:1 molar ratio, [Salt2]:[CNS] 8:1 molar ratio, letters (a,b)—peaks height.

	r Values		
	**λ 250–270 nm**	**λ > 270 nm**	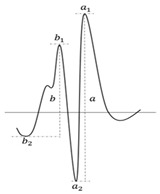
HSA	0.65	2.00	
Salt2-HSA_complex_	0.84	2.24	
AGP	0.77	0.43	
Salt2-AGP_complex_	0.62	0.49	
HGG	1.00	0.80	
Salt2-HGG_complex_	0.55	0.77	
CNS	0.92	0.64	
Salt2-CNS_complex_	0.67	0.72	

**Table 4:** Stern–Volmer constants (K_S-V_) and bimolecular fluorescence quenching rate constants (k_q_) of the studied systems (λ_ex_ 275 nm and λ_ex_ 295 nm).

	λ_ex_ 275 nm		λ_ex_ 295 nm	
	(K_s-v_ ·10^4^) ± SD * [mol·L^−1^]	(k_q_ ·10^12^) ± SD * [mol^−1^·L·s^−1^]	(K_s-v_ ·10^4^) ± SD * [mol·L^−1^]	(k_q_ ·10^12^) ± SD * [mol^−1^·L·s^−1^]
Salt2-HSA_complex_	2.01 ± 0.04	3.35 ± 0.06	2.27 ± 0.02	3.79 ± 0.04
Salt2-AGP_complex_	8.13 ± 0.15	13.55 ± 2.43	7.28 ± 2.13	12.13 ± 3.54
Salt2-HGG_complex_	1.27 ± 0.02	2.08 ± 0.04	1.83 ± 0.03	3.00 ± 0.05
Salt2-CNS_complex_	1.79 ± 0.03	2.84 ± 0.05	2.06 ± 0.07	3.43 ± 0.12

* standard deviation.

**Table 5:** The binding parameters for Salt2-HSA, Salt2-AGP, Salt2-HGG and Salt2-CNS complex.

	λ_ex_ 275 nm		λ_ex_ 295 nm	
	(K_a_ ·10^4^) ± SD * [mol·L^−1^]	n ± SD *	(K_a_ ·10^4^) ± SD * [mol·L^−1^]	n ± SD *
(Salt2-HSA)_complex_	1.98 ± 0.07	0.97 ± 0.04	2.33 ± 0.18	0.97 ± 010
(Salt2-AGP)_complex_	5.04 ± 0.25	0.92 ± 0.07	6.28 ± 0.11	0.95 ± 0.04
(Salt2-HGG)_complex_	3.45 ± 0.29	0.97 ± 0.12	3.28 ± 0.10	0.96 ± 0.12
(Salt2-CNS)_complex_	0.94 ± 0.04	0.99 ± 0.05	0.91 ± 0.43	1.99 ± 1.19

* standard deviation.

**Table 6:** The mean residue ellipticity [Θ_MRW_] and the percentage (%) content of the secondary structure elements of HSA and AGP based on the Yang’s and Reed’s reference model, respectively.

	[Θ]_MRW_ at 208.8 nm [mdeg·cm^2^·dmol^−1^]	[Θ]_MRW_ at 222 nm [mdeg·cm^2^·dmol^−1^]	% α-helix	% β-sheet	% Turn	% Random
HSA ^a^	−21,690.171	−20,413.846	37.0	10.3	21.5	31.2
Salt2-HSA_complex_^a^	−21,794.444	−21,690.171	37.4	10.1	21.7	30.9
AGP ^b^	-	−9130.429	16.5	83.5	-	-
Salt2- AGP_complex_^b^	-	−9027.758	16.4	85.6	-	-

^a^ Yang’s reference model; ^b^ Reed’s reference model.

**Table 7:** The percentage of displacement of dansylated amino acids from the HSA molecule in the presence of Salt2 with increasing concentration; [HSA]=[dGly]=[dPhe]=[Salt2] 5 × 10^−6^ mol·L^−1^; λ_ex_ 350 nm.

C_Salt2_ [mol·L^−1^]	[HSA]:[dGly] Molar Ratio	[HSA]:[dPhe] Molar Ratio
	1:1	
	Percentage of Displacement [%]	
0	-	-
3.3 × 10^−5^	65.55	46.11

**Table 8:** The percentage of displacement of QR from the AGP molecule in the presence of Salt2 with increasing concentration; [AGP]=[QR]=[Salt2] 3 × 10^−6^ mol·L^−1^; λ_ex_ 500 nm.

C_Salt2_ [mol·L^−1^]	[AGP]:[QR] 1:0.5 Molar Ratio	[AGP]:[QR] 1:1 Molar Ratio
	Percentage of Displacement [%]	
0	-	-
4.8 × 10^−5^	66.5	69.6

## Data Availability

Samples of the compounds are available from the authors.

## Supplementary Materials

The following supporting information can be downloaded at: https://www.mdpi.com/article/10.3390/molecules28020698/s1, Figure S1: Emission fluorescence spectra of human serum albumin (HSA) in the presence of Salt2 at Salt2:HSA 0:1 and 7:1 and Salt2:HSA 0:1 and 6:1 molar ratios at (**a**) λ_ex_ 275 nm and (**b**) λ_ex_ 295 nm, respectively; Figure S2: Emission fluorescence spectra of α1-acid glycoprotein (AGP) in the presence of Salt2 at Salt2:HSA 0:1 and 8:1 and Salt2:HSA 0:1 and 7:1 molar ratios at (**a**) λ_ex_ 275 nm and (**b**) λ_ex_ 295 nm, respectively; Figure S3: Emission fluorescence spectra of human gamma globulin (HGG) in the presence of Salt2 at Salt2:HSA 0:1 and 7:1 and Salt2:HSA 0:1 and 6:1 molar ratios at (**a**) λ_ex_ 275 nm and (**b**) λ_ex_ 295 nm, respectively; Figure S4: Emission fluorescence spectra of control normal serum (CNS) in the presence of Salt2 at Salt2:HSA 0:1 and 7:1 and Salt2:HSA 0:1 and 6:1 molar ratios at (**a**) λ_ex_ 275 nm and (**b**) λ_ex_ 295 nm, respectively.
