# Supplementary material for: Spectroscopic Studies of Quinobenzothiazine Derivative in Terms of the In Vitro Interaction with Selected Human Plasma Proteins: Part 2
Source: Molecules. 2023 Jan 10;28(2):698. doi: 10.3390/molecules28020698 (PMC9865466; doi:10.3390/molecules28020698)
Supplement: Supplementary file 1 [file molecules-28-00698-s001.zip › molecules-2083109-supplementary.pdf]

Supplementary materials

Spectroscopic Studies of Quinobenzothiazine Derivative in Terms of the In Vitro Interaction with Selected Human Plasma Proteins. Part 2

Aleksandra Owczarzy [1], Wojciech Rogóż [1], Karolina Kulig [1], Jadwiga Pożycka [1], Andrzej Zięba [2] and Małgorzata Maciążek-Jurczyk [1,*]

[1] Department of Physical Pharmacy, Faculty of Pharmaceutical Sciences in Sosnowiec, Medical University of Silesia, 40-055 Katowice, Poland

[2] Department of Organic Chemistry, Faculty of Pharmaceutical Sciences in Sosnowiec, Medical University of Silesia, 40-055 Katowice, Poland

* Correspondence: mmaciazek@sum.edu.pl; Tel.: +48-32-364-1582

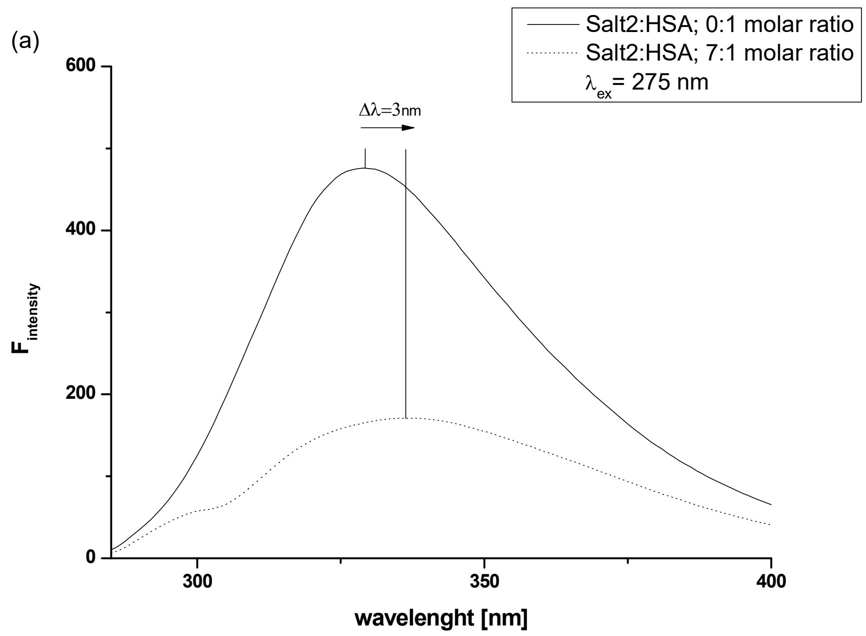

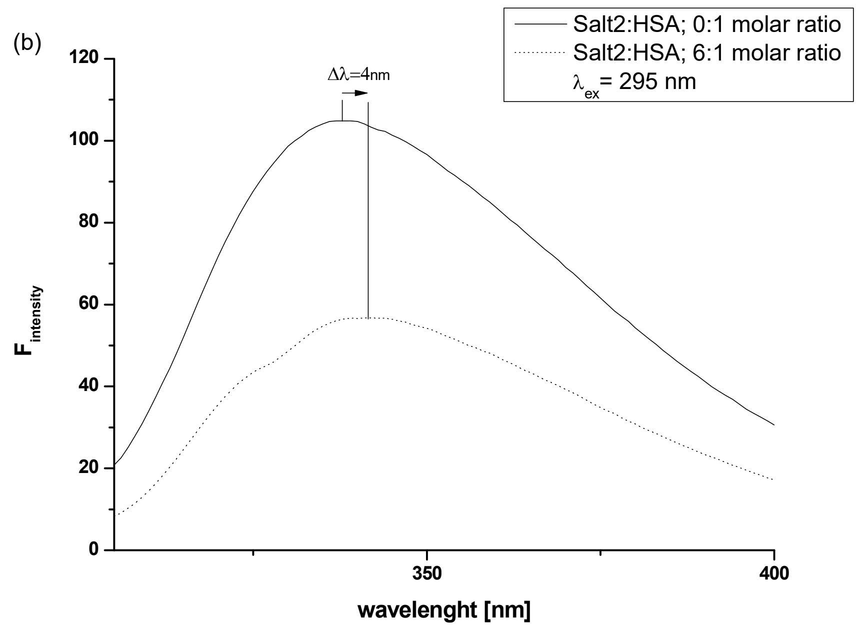

Figure S1. Emission fluorescence spectra of human serum albumin (HSA) in the presence of Salt2 at Salt2:HSA 0:1 and 7:1 and Salt2:HSA 0:1 and 6:1 molar ratios at a) λ_{ex} 275 nm and b)λ_{ex}295 nm, respectively.

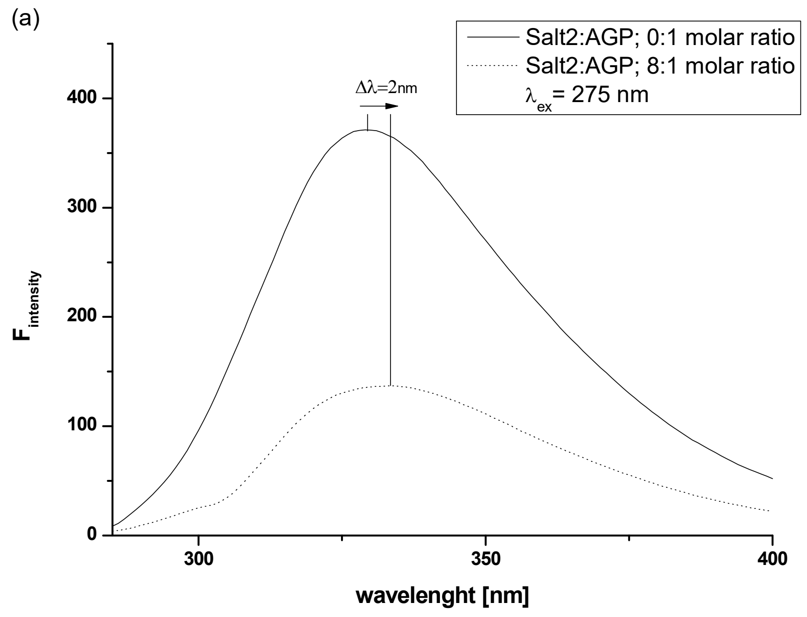

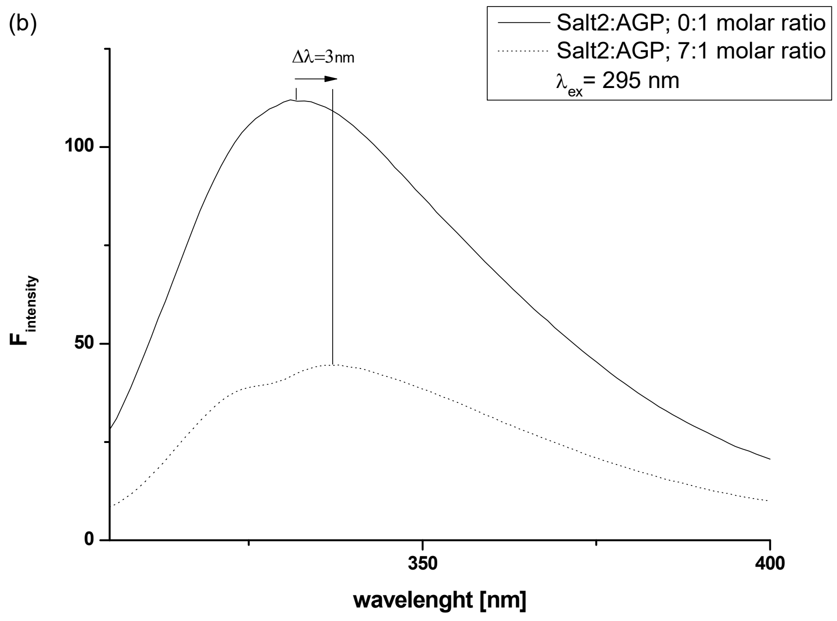

Figure S2. Emission fluorescence spectra of α_1-acid glycoprotein (AGP) in the presence of Salt2 at Salt2:HSA 0:1 and 8:1 and Salt2:HSA 0:1 and 7:1 molar ratios at a) λ_{ex} 275 nm and b) λ_{ex} 295 nm, respectively.

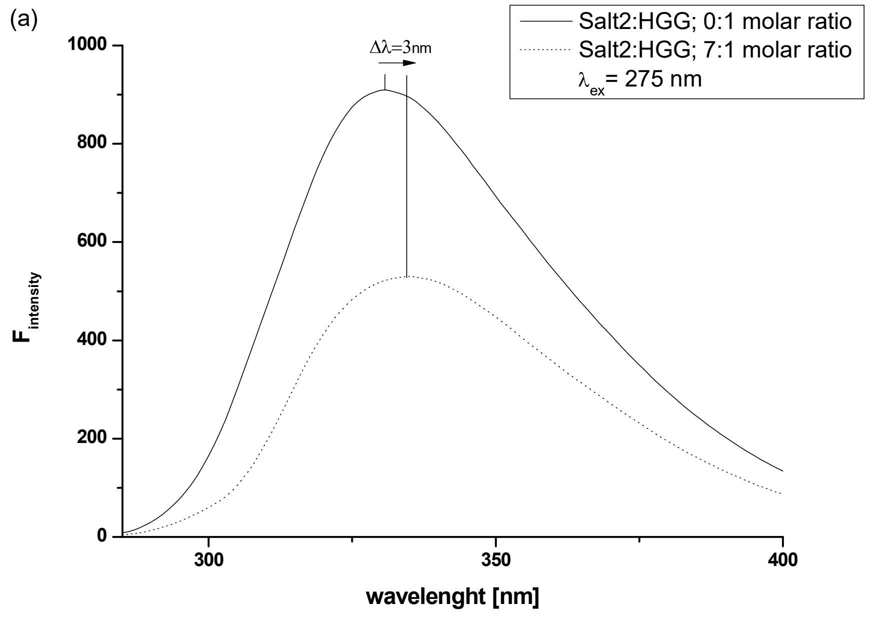

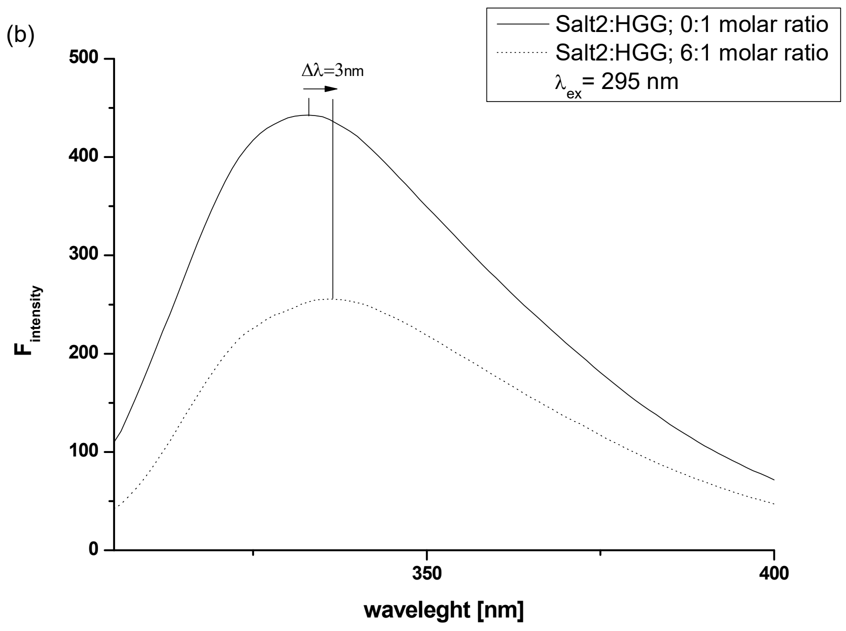

Figure S3. Emission fluorescence spectra of human gamma globulin (HGG) in the presence of Salt2 at Salt2:HSA 0:1 and 7:1 and Salt2:HSA 0:1 and 6:1 molar ratios at a) λ_{ex} 275 nm and b) λ_{ex} 295 nm, respectively.

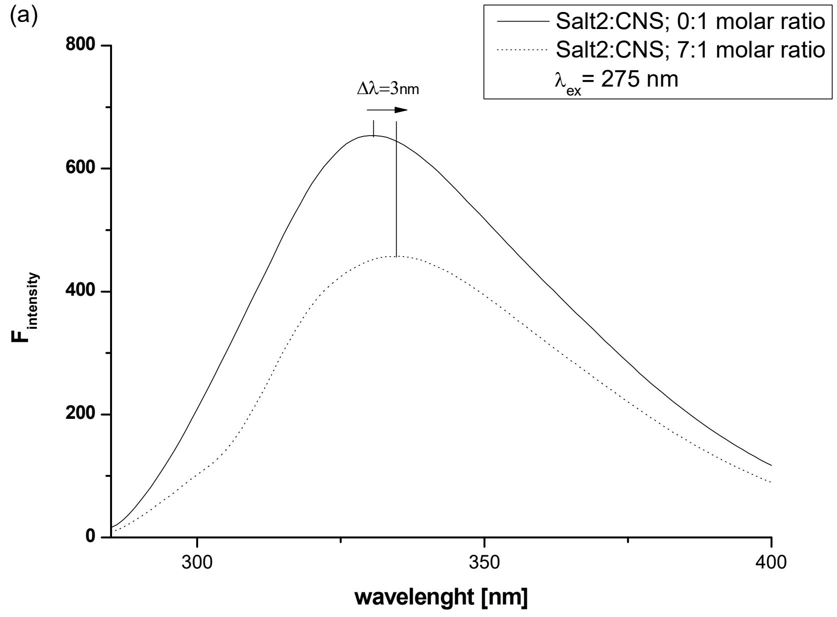

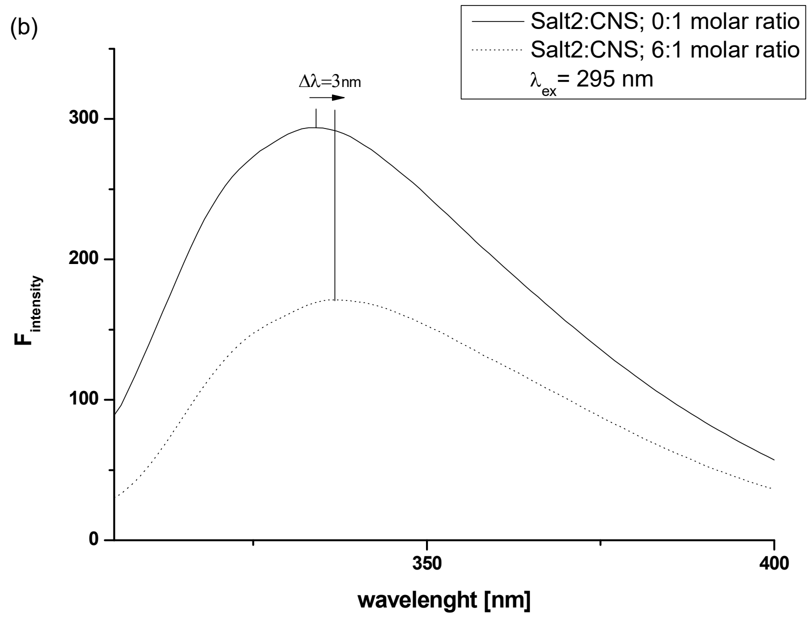

Figure S4. Emission fluorescence spectra of control normal serum (CNS) in the presence of Salt2 at Salt2:HSA 0:1 and 7:1 and Salt2:HSA 0:1 and 6:1 molar ratios at a) λ_{ex} 275 nm and b) λ_{ex} 295 nm, respectively.
